# Evaluating the impact of discordant and missing demographic information on population health assessments using linked electronic health records and Census Bureau microdata

**DOI:** 10.1371/journal.pdig.0001289

**Published:** 2026-03-17

**Authors:** Derek Ouyang, Aubrey Limburg, David H. Rehkopf, Jacob Goldin, Robert L. Phillips, Victoria Udalova, Daniel E. Ho

**Affiliations:** 1 Stanford Law School, Stanford, California, United States of America; 2 U.S. Census Bureau, Suitland, Maryland, United States of America; 3 School of Medicine, Stanford University, Stanford, California, United States of America; 4 University of Chicago Law School, Chicago, Illinois, United States of America; 5 American Bar Foundation, Chicago, Illinois, United States of America; 6 American Board of Family Medicine, Lexington, Kentucky, United States of America; 7 Department of Computer Science, Stanford University, Stanford, California, United States of America; 8 Department of Political Science, Stanford University, Stanford, California, United States of America; The University of Arizona, UNITED STATES OF AMERICA

## Abstract

Administrative records are increasingly being used to study population-level outcomes, despite high rates of missingness and discrepancies (*i.e.*, discordance) in demographic identifiers across different sources of data, which could reduce the quality of such assessments. Few studies have evaluated the relationship between these phenomena in administrative records and downstream impacts on assessments in consequential domains such as healthcare. We characterize patterns of discordance and missingness of race and ethnicity in electronic health records (EHR; 2010–2021) derived from the American Board of Family Medicine’s primary care registry, linked at the individual-level to restricted U.S. Census Bureau microdata (2000, 2010, 2020 Census; American Community Survey 2005–2022). Among 5.86 million linked patients, 19.3% were missing race and ethnicity information in EHRs, and 8.0% had race and ethnicity information that was recorded discordantly between the two sources, with the lowest discordance for White, Black, and Asian patients and the highest for American Indian and Alaska Native, Native Hawaiian and Pacific Islander (NHPI), and Multiracial patients. Missingness and discordance impacted estimation of group differences for all 50 health outcomes we consider, particularly for smaller racial/ethnic groups, such as a 24 percent change in NHPI Type 2 diabetes diagnosis rates. Our research has three major implications for the work of government agencies, academics, clinicians, and other stakeholders interested in utilizing EHRs for research purposes. First, we demonstrate how the quality of demographic data in administrative records can be comprehensively assessed, which previously has not been possible due to limitations in data access and linkage. Second, we systematically evaluate the impact of discordant and missing demographic information on our ability to accurately estimate disease prevalence. Third, we underscore the importance of evaluating discordance of demographic information both within and across different administrative domains.

## Author summary

Population-level assessments in consequential domains such as healthcare depend on large, high-quality administrative data. However, discordance and missingness of demographic information across records can distort analyses conducted by researchers and policymakers. We provide robust and comprehensive evidence and characterization of these patterns through a dataset of 5.86 million patients in the United States with linked information from electronic health records and restricted U.S. Census Bureau microdata. In particular, we demonstrate how these data quality issues can affect estimation of consequential group-level health outcomes, such as Type 2 diabetes diagnosis rates. Discordance and missingness are widespread and highly concentrated in specific administrative settings like primary care clinics, creating the potential for error at every geographic scale of assessment. However, much can be done to diagnose and mitigate discordance and missingness, particularly at the point when demographic information is collected. With more complete and concordant demographic information and improved data quality in electronic health records and other administrative records, government agencies, academics, and practitioners can more accurately measure and address health challenges.

## 1. Introduction

Government agencies, academics, and practitioners are increasingly leveraging large administrative datasets with individually recorded demographic information to conduct population-level assessments. Across domains such as voting [1], criminal sentencing [2], and mortgage lending [3], such assessments often assume that available demographic information is accurately collected, standardized, and consistent over time. In settings where such demographic information is missing, researchers and policymakers sometimes seek to recover missing information through statistical methods [4–6], which themselves are either trained on or derived from other available demographic information which is also assumed to be accurate.

Few studies or assessments involving estimation of group-level outcomes have directly considered whether the demographic variables contained within their datasets [7], or otherwise relied upon for statistical imputation [8], might be less accurate than assumed. Nor have most studies considered whether the analyses’ substantive findings and/or statistical confidence might be sensitive to variability in this information. This is in spite of longstanding and growing evidence, from sociology to demography to clinical research, that socially meaningful characteristics like race and ethnicity—our focus for this study—recorded for the same individual across administrative datasets or across time can be discordant, or non-matching, for a variety of reasons [9,10].

One limitation to evaluating the veracity of demographic information in any particular study is the lack of a secondary administrative dataset to evaluate such assumptions. Such a companion dataset, with similar coverage and availability as the first, could be used to perform (verifiably high-fidelity) record linkage, followed by comparison of side-by-side labels for individuals. It is with this context that our study makes two key contributions. First, we fully characterize discordance and missingness of race/ethnicity labels as recorded in electronic health records (EHR) for millions of primary care patients in the United States (U.S.), linked to U.S. Census Bureau restricted survey and census microdata sources [11]. Our research expands on previous work in this area [12] by scaling our evaluation to millions of individuals and utilizing linked data from two national sources: EHRs and Census Bureau-collected data. Second, we evaluate how patterns of discordance and missingness impact population health assessments. In particular, by comparing observations for the same individuals collected across two widely different settings—decentralized primary care practices versus a federal statistical system—we can begin to distinguish between intra-domain (*e.g.*, within Census Bureau instruments) and inter-domain (*e.g.*, Census Bureau vs. EHR) discordance. Our paper proceeds with a review of related literature on missing and discordant demographic information, continues with a presentation of our data, methods, and results, and concludes with implications of our findings for government agencies, academics, and practitioners conducting population-level assessments.

## 2. Background

### A. Missing demographic information

Although health records are increasingly being used to study health outcomes [13–15], they typically contain more information about services, treatments, and outcomes (*e.g.*, diagnoses, medications, healthcare utilization) than on demographic attributes [16–18]. Relying on only individuals with recorded demographics could lead to misestimation of group-level outcomes, given high (and likely non-random) rates of missing demographic information [19–22]. For example, while Medicaid enrollment data have been used to assess a variety of health outcomes [23–26], roughly one in five beneficiaries are missing race/ethnicity information, with substantial variation across states [27]. Linking Medicaid records to Census Bureau microdata has shown that state-level racial and ethnic mortality estimates differ when based only on Medicaid data, relative to linked data [28].

When direct record linkage is not possible, researchers and practitioners often turn to statistical methods to recover missing demographic information [29], such as multiple imputation [30]. Other common approaches, such as Bayesian Improved Surname Geocoding (BISG) and its counterpart which incorporates first names [31–33], leverage conditional probabilities of race and ethnicity given name and geography from large administrative datasets. They have been widely applied in consumer lending [6,34,35], voting [1,36], evictions [37], insurance [38], health [39–42], and taxation [40,41,43], and have also been extended to disaggregated race categories [44].

We underscore two takeaways from this review of the missingness literature. First, the estimation of outcomes is sensitive to how missing demographic data are handled, whether through exclusion, imputation, or record linkage. Linkage may offer greater accuracy but is often prohibitive due to data availability and/or privacy concerns. Our study contributes a geographically comprehensive demonstration of how individuals with missing race/ethnicity information differ in their racial and ethnic makeup, relative to those without missing information, as well as how the outcomes of population health assessments are sensitive to this missingness.

Second, recovery of missing information assumes a reliable external source of demographic information, whether from a linked dataset or from administrative data which form the basis of tools like BISG. Yet, even analyses using the non-missing information in the primary dataset itself are also predicated on an assumption of reliability. Each of these distinct sources of demographic information may be impacted by a separate data quality issue, discordance, to which we now turn.

### B. Discordant demographic information

Relative to missingness, discordance of demographic information between sources is considerably less studied in the literature [12]. Previous research using linked Medicaid and Census Bureau microdata identified individual states with upwards of 10% discordance between race and ethnicity information for individuals who had a recorded race and ethnicity in both sources, and observed that smaller racial and ethnic groups are more likely to have discordant labels [28,45]. Discordance may also exist across Census Bureau census and survey data [10,46], across healthcare records [47–49], and between healthcare records and self-reported surveys [50–54].

Past work has examined the possible drivers of discordance in demographic reporting across different sources. First, in the healthcare domain, studies have found heterogeneity in the options patients have when asked to select their race and ethnicity in a patient intake form [19]. Patients who are prevented from selecting their preferred race and ethnicity, or multiple preferred races, in one dataset may appear to respond discordantly across sources. EHR systems also differ in how they map free-text responses and when they adopt changes in federal standards [55]. We note that federal standards themselves have changed multiple times over the decades, often in direct response to demographic and political changes in American society [56], including 1997 revisions that disaggregated the Asian and Pacific Islander (API) category into two separate categories [57] and, most recently, 2024 revisions that combined racial and ethnic categories while significantly increasing the level of disaggregation required in information collection by federal agencies [58]. Federal revisions constitute a potential root cause of discordance if and when agencies adopt new standards at different times. Even federal agencies directly subject to revisions may continue to make use of older racial and ethnic categories. For instance, the Census Bureau’s published 2010 surname tables, widely used in BISG imputation, do not reflect the 1997 revisions and still make use of the 1977 API category [59], meaning that most imputations by researchers and practitioners do not distinguish Asian from Native Hawaiian and Other Pacific Islander (NHPI). Downstream adoption of federally revised standards by state and local agencies, as well as private entities like healthcare systems, vary even more widely in their degree and timing, further increasing the opportunities for discordant standards to drive discordant records.

Second, the demographic information documented for an individual may not always be self-reported. A study linking criminal justice administrative records, in which race and ethnicity are often determined by justice agency personnel, and Census Bureau microdata estimated discordance rates ranging from 17% in court records to 10% in state prison records [60]. The authors estimate that, as a result of these discordances, federal incarceration rates may be substantially underestimated for White, Black, and American Indian and Alaska Native populations. In healthcare, studies have documented cases of clinicians or receptionists subjectively assigning race and ethnicity on behalf of a patient, as opposed to patient self-reporting [61]. Such third-party reporting has the potential to affect data quality and quality of care.

Third, as a social category, race and ethnicity are fluid, even though much population work has not treated them that way [62]. The variability within individuals over time is substantial, *i.e.*, around 6% when comparing responses to questions on race and ethnicity between the 2000 and 2010 Decennial Census [10]. Researchers have argued that discordance between administrative records collecting data at different periods of time can’t be entirely attributed to error, and is at least in part due to changing self-identification [9], as well as to the context around which questions are asked, which differ substantially between a medical clinic [*e.g.*, 63] and, say, a census form.

### C. Electronic health records and population health assessments

Few studies have evaluated discordance in a large population setting, due to the challenge of record linkage across disparate administrative sources. Fewer still have comprehensively characterized the relationship between missingness, discordance, and downstream impacts on evaluations of health outcomes. One existing study examines discordance in a single state’s public integrated healthcare system by linking individual health records to restricted American Community Survey (ACS) microdata [64]. This research found high rates of concordance for those who identified as Black, White, or Hispanic. However, rates of concordance were considerably lower for other groups. Our study builds on this work by examining discordance across all fifty states, utilizing a private and decentralized system of primary care clinics in which we might expect even greater risk of discordance, leveraging record linkage to both ACS and a larger universe of decennial census respondents, and incorporating a wide range of health outcomes from the EHRs to demonstrate the degree to which discordant demographic information can affect the quality of population health assessments.

## 3. Methods

### A. Data

This research is the product of a collaboration between the American Board of Family Medicine (ABFM), Stanford University, and the Enhancing Health Data (EHealth) Program at the U.S. Census Bureau. The purpose of this collaboration is to conduct novel linkages between EHR data and restricted Census Bureau microdata as a means of producing high quality statistics and conducting health research that marries clinical and social indicators in novel ways. Creation of blended data using EHR data and Census Bureau microdata expands the type and quality of data products and research that can be created. This allows the public and policymakers to improve decision making by implementing policies that directly impact individuals’ access to care, quality of care, and health outcomes.

For this retrospective secondary data analysis, we relied on data from two main sources [11]. The first and primary source of data consisted of EHR data from the American Family Cohort, a research dataset derived from the PRIME Registry by ABFM between 2010 and 2021 [65,66]. The PRIME Registry is the largest outpatient clinical registry open to all primary care clinicians in the U.S., includes over 1,000 small primary care practices, and captures information collected during clinical encounters for over 7 million patients residing in the U.S., including sociodemographic characteristics (*e.g.*, age, sex, race/ethnicity), residential location, and clinical history.

EHR data were brought into the secure Census Bureau IT environment and processed through the Person Identification Validation System (PVS) [67]. The PVS uses person-level information, including Social Security number (SSN), date of birth, name, sex, and address information (but not race/ethnicity), to match individual records to an internal reference file. If either a deterministic (primary) or a probabilistic (fallback) match is made between the EHR data and the Census Bureau reference file, the record is assigned a Protected Identification Key (PIK), which is a unique anonymized person-level identifier that is used across all demographic data sources at the Census Bureau. Once a PIK has been assigned (or not), SSN, name, and address are removed from the file. Therefore, researchers did not have access to identifying patient information during analysis but rather relied on anonymized PIKs.

In the EHR data, 97.7% of all eligible patients received a PIK. Prior studies have more thoroughly evaluated assignment rates and false match rates in the PVS [68,69], and while some studies have found differences in PIK assignment rates by demographic characteristics including race/ethnicity [28], a recent study [11] assessed the same EHR-based PIK assignment procedure as our study for a smaller subset of patients and found insubstantial disparities in assignment rates by race/ethnicity (from the standpoint of EHR-recorded race/ethnicity).

Once PIKs were assigned, EHR data were linked to the second source of data, Census Bureau microdata including decennial census (2000, 2010, 2020) and American Community Survey (ACS) 1-year microdata (2005–2022). Of those who received PIKs, 93.9% (n = 5.86 million) had valid race and ethnicity information in any Census Bureau microdata source. Subsequent analyses focus exclusively on patients who received PIKs and were successfully matched to the Census Bureau microdata sources to obtain race and ethnicity information. These data were first accessed for the study on September 25, 2023.

### B. Ethics statement

Secure disclosure of EHR data to the Census Bureau satisfies the data owner’s obligation to protect these data as required by the Health Insurance Portability and Accountability Act (HIPAA). Individual patient consent was not obtained for this study, as the research involved the analysis of anonymized electronic health records, and Title 13 of the U.S. Code authorizes the Census Bureau’s record linkage activities. The Census Bureau does inform respondents that their information will be used only for statistical purposes, and it informs the public about its record linkage activities (including how they are conducted, the purposes for which they are conducted, and the benefits derived from them) through its System of Records Notices published in the Federal Register, and also through information posted on its website. The record linkages are strictly confidential under Title 13 and can only be used for statistical purposes that help the Census Bureau conduct its Title 13 authorized work.

### C. Measures

#### i. Race and ethnicity.

There is considerable variation in how race/ethnicity information is collected across different healthcare systems [19]. Without standardization, particularly for open text responses, discordance across health records would be far more ubiquitous. Our aim, instead, is to assess the degree to which discordance exists after reasonable steps of standardization, as researchers and population health practitioners would have performed on their data prior to analysis. As such, we harmonize race/ethnicity information in our EHR data for comparison with Census Bureau microdata using the following standard categories established by the Office of Management and Budget (OMB) in 1997 [57]: Hispanic or Latino (Hispanic), non-Hispanic White (White), non-Hispanic Black or African American (Black), non-Hispanic American Indian and Alaska Native (AIAN), non-Hispanic Asian (Asian), non-Hispanic Native Hawaiian and Other Pacific Islander (NHPI), and non-Hispanic Multiple Races (Multiracial). We define discordance as non-matching selections between EHR and Census Bureau microdata, after harmonization to these standard categories. For those who identified as Multiracial in Census Bureau microdata, we can also observe their distinct race selections, and so if one of these distinct races were to match to a single race reported by the same individual in EHR data, we define this as partial concordance. More details can be found in Appendix A in S1 Text.

Census Bureau microdata includes separately collected race/ethnicity information from the 2000, 2010, and 2020 Census and 2005–2022 ACS 1-year microdata, all of which were collected in accordance with OMB standards. All survey responses are collected at the household level, and a single person responds on behalf of the entire housing unit. There were instances in which individuals had multiple observations across sources and years. In those cases, we prioritized the most recent reporting of race/ethnicity within a data source and prioritized the decennial census over the ACS (though some analyses explicitly compare multiple observations across sources and years). Before prioritizing a given observation, we omitted some observations entirely if: *(1)* race/ethnicity responses were imputed or allocated, rather than reported by the individual or a household member; or *(2)* an individual had multiple non-matching race/ethnicity responses within a given data source and year (which can be indicative of record linkage error), in which case all of their observations for that source and year were omitted.

#### ii. Health outcomes.

The EHR data include specific health outcomes, including diagnoses and routine healthcare events, stemming from clinical visits for all patients during the period from 2010 to 2021 and recorded by individual practices. The health outcome codes are available in International Classification of Diseases Ninth Revision (ICD-9) and Tenth Revision (ICD-10) and Systematized Nomenclature of Medicine - Clinical Terms (SNOMED CT) formats. We mapped ICD-9 codes and SNOMED CT codes to their ICD-10 equivalent and collapsed all ICD-10 codes to their first 3 digits, which describe the general disease or category. For example, a patient may be recorded with E11.42 (Type 2 diabetes with polyneuropathy), while another patient may be recorded with E11.65 (Type 2 diabetes with hyperglycemia), but for the purposes of our analysis, both patients were identified as having a record of E11 (Type 2 diabetes). For each patient, we determined whether they ever had each unique 3-digit outcome, then determined the top 50 most prevalent outcomes across all patients. See Table A in S1 Text for a list of these codes and their descriptions. We note that these prevalence estimates do not necessarily reflect prevalence of the underlying health conditions in the U.S. population. Our measure is more a reflection of care-seeking behavior than actual disease prevalence, and may also interact with discordance itself in subtle and important ways.

#### iii. Additional covariates.

The EHR data also include: patient date of birth, from which we derive age; patient sex, which we code into the categories male and female; and patient residential address, which we clean and geocode (*i.e.*, omit multiple conflicting or unresolved addresses) to census block groups for the purpose of obtaining a 2022 Social Vulnerability Index (SVI) score from the Centers for Disease Control and Prevention [70], and to ZIP codes for the purpose of obtaining a 2010 Rural-Urban Commuting Area (RUCA) code from the U.S. Department of Agriculture [71]. We also retain an anonymized practice-level identifier for each patient, allowing us to group patients by practice to conduct practice-level regressions.

### D. Statistical methods

Our primary outcomes of discordance and missingness are direct descriptive statistics based on the proportions of patients who report race/ethnicity discordantly or with missingness, as observed from the standpoint of any particular reference information source. All analyses were conducted using R version 4.2.

We use the additional patient covariates to conduct two-sample tests for equality of proportions (with continuity correction) for all 39 pairwise comparisons across discordant, concordant, and non-reporting groups, applying a Bonferroni correction with a family-wise error rate of 0.05 to adjust our confidence intervals.

For our analysis of practice-level effects, we calculate practice-level attributes and fit three simple linear regression models on the set of 1,290 unique practices.

For our analysis of the impact of discordance and missingness on health outcomes, we fit separate weighted logistic regression models for each combination of racial/ethnic group and health outcome to estimate the difference in group-level prevalence as estimated using EHR data versus Census Bureau microdata, with Wald-type confidence intervals constructed using the delta method. Given 350 total comparisons, we apply a Bonferroni correction with a family-wise error rate of 0.05 to adjust our confidence intervals.

For our deeper analysis of Type 2 diabetes, in which we consider 19 distinct patterns of concordance, discordance, and missingness that shape changes in estimated group-level prevalences, we construct confidence intervals for prevalence rates using the Wilson Score method (with continuity correction), and apply a Bonferroni correction with a family-wise error rate of 0.05 for adjustment.

## 4. Results

### A. Discordance and missingness

#### i. Comparison of EHR and Census Bureau race/ethnicity.

**Fig 1:**
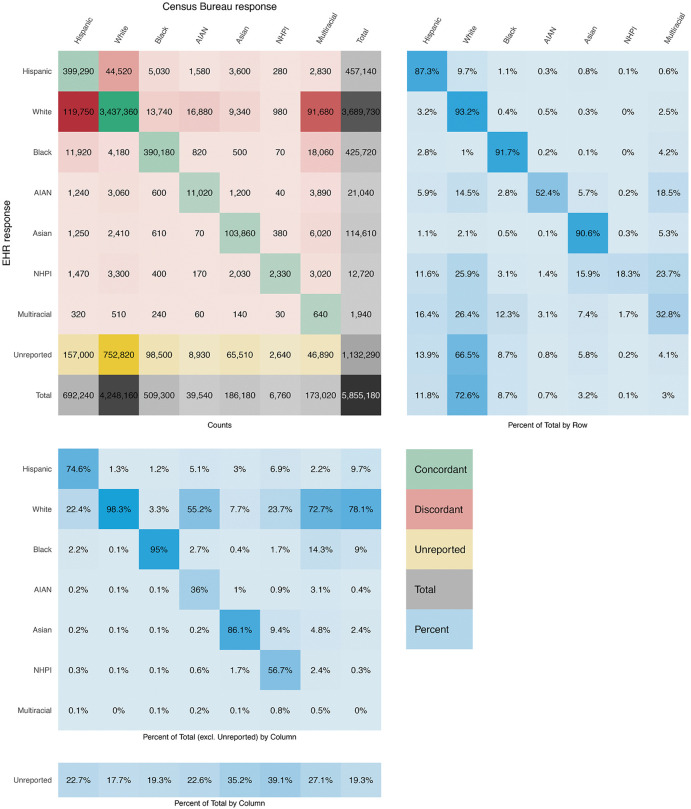
Matrix comparing race/ethnicity in Census Bureau microdata (grouped by column) relative to race/ethnicity in EHR data (grouped by row), for 5.86 million individuals. (Top left) Counts of individuals. (Top right) Percentages which sum to 100 by row and represent the distribution of those individuals in an EHR racial/ethnic group as recorded in Census Bureau microdata. (Bottom left) Percentages which sum to 100 by column (excluding Unreported) and represent the distribution of those individuals in a Census Bureau racial/ethnic group as recorded in EHR data; Unreported is presented separately as a percent of total individuals in a Census Bureau racial/ethnic group. Color scales are for illustration only. Source: Electronic health record (EHR) data (2010–2021); 2000, 2010, and 2020 Census; American Community Survey (2005–2022). Note: The Census Bureau has reviewed this data product to ensure appropriate access, use, and disclosure avoidance protection of the confidential source data used to produce this product (Data Management System (DMS) number: P-7527965, Disclosure Review Board (DRB) approval number: CBDRB‑FY24‑0453). Discrete Gaussian noise was applied to all unweighted counts according to U.S. Census Bureau disclosure protocols to preserve data privacy.

Fig 1 visualizes 5.86 million patients for whom linkage was successful between EHR data and Census Bureau microdata, and valid race/ethnicity data was available in Census Bureau microdata. We highlight three insights below.

First, 8.0% (378,060/5.86M) of all individuals who have race/ethnicity information in both sources are recorded discordantly. From the standpoint of either information source, the three smallest racial/ethnic groups (AIAN, NHPI, and Multiracial) exhibit the highest discordance, while the White, Black, and Asian groups exhibit low discordance. Ultimately, of all discordant patients, from the standpoint of EHR-recorded race/ethnicity, 66.7% (252,360/378,060) are White, 15.3% are Hispanic, and 0.3% are Multiracial. But from the standpoint of Census Bureau-recorded race/ethnicity, 15.3% are White, 35.9% are Hispanic, and 33.2% are Multiracial (see Table 1). In effect, EHR data reflect a less heterogeneous population (21.9% non-White) than Census Bureau microdata (27.4% non-White).

**Table 1:** Percent of patients recorded discordantly by race/ethnicity, as distinguished between EHR-recorded race/ethnicity and Census Bureau-recorded race/ethnicity.

		Discordant	
		EHR-recorded race/ethnicity	Census Bureau-recorded race/ethnicity
	Total	378,060	
Race/Ethnicity	Hispanic	57,840 (15.3%)	135,940 (35.9%)
	White	252,360 (66.7%)	57,980 (15.3%)
	Black	35,540 (9.4%)	20,620 (5.4%)
	AIAN	10,020 (2.6%)	19,580 (5.2%)
	Asian	10,750 (2.8%)	16,810 (4.4%)
	NHPI	10,380 (2.8%)	1,780 (0.5%)
	Multiracial	1,300 (0.3%)	125,500 (33.2%)

Source: Electronic health record (EHR) data (2010–2021); 2000, 2010, and 2020 Census; American Community Survey (2005–2022).

Note: The Census Bureau has reviewed this data product to ensure appropriate access, use, and disclosure avoidance protection of the confidential source data used to produce this product (Data Management System (DMS) number: P-7527965, Disclosure Review Board (DRB) approval number: CBDRB‑FY24‑0453). Discrete Gaussian noise was applied to all unweighted counts according to U.S. Census Bureau disclosure protocols to preserve data privacy.

Second, the single lowest concordance rate we observe is for Multiracial patients in either EHR data or Census Bureau microdata: only 0.5% (640/127,160) of them are recorded as Multiracial in both sources, with far more recorded by the Census Bureau. Considering partial concordance, only 5.0% of those who are recorded as Multiracial in at least one source are fully discordant (*i.e.*, the single race recorded in EHR data does not match any of the distinct races comprising Multiracial in Census Bureau microdata), a rate more consistent with what we observe for White and Black individuals. If we allow for partial concordance, the overall discordance rate in our full cohort decreases from 8.0% to 5.4%.

Third, regarding missingness, 19.3% (1.13M/5.86M) of all patients report their race/ethnicity to the Census Bureau, but do not have recorded race/ethnicity in EHR data. From the standpoint of Census Bureau race/ethnicity, EHR missingness ranges from a low of 17.7% (752,820/4.25M) for White individuals to 35.2% (65,510/186,180) and 39.1% (2,640/6,760) for Asian and NHPI individuals, respectively. This wide variation in missingness rates across groups is otherwise unobservable to researchers with access to EHR data alone.

#### ii. Demographic characteristics.

Table 2 compares demographic characteristics across three different patient groups: those who are recorded concordantly across EHR and Census Bureau microdata, those who are recorded discordantly, and those who do not report in EHR data. Notably, concordant patients are 13.6% [13.4-13.8] more likely than discordant patients to be 65 and older, 6.0% [5.9-6.2] less likely than nonreporting patients to live in metropolitan areas, and 8.7% [8.5-9.0] less likely than discordant patients to live in areas with higher levels of social vulnerability (SVI of 75+).

**Table 2:** Percent of patients in concordant, discordant, and non-reporting groups, by age, sex, and tiers of SVI and RUCA codes as determined using patient address.

		Concordant	Discordant	Nonreporting
	Total	4,344,600	378,060	1,132,220
Age	Less than 18	305,350 (7.0%)	46,830 (12.4%)	84,990 (7.5%)
	18–64	2,491,460 (57.3%)	248,000 (65.6%)	715,970 (63.2%)
	65 and over	1,547,780 (35.6%)	83,240 (22.0%)	331,270 (29.3%)
Sex	Male	1,944,320 (44.8%)	163,120 (43.2%)	509,220 (45.0%)
	Female	2,397,990 (55.2%)	214,560 (56.8%)	619,120 (54.7%)
Social Vulnerability Index (SVI)	[0,25]	987,490 (22.7%)	67,420 (17.8%)	247,200 (21.8%)
	(25,50]	1,089,060 (25.1%)	78,480 (20.8%)	246,370 (21.8%)
	(50,75]	992,050 (22.8%)	88,410 (23.4%)	245,270 (21.7%)
	(75,100]	642,060 (14.8%)	88,920 (23.5%)	205,250 (18.1%)
Rural-Urban Commuting Area Code (RUCA)	Metropolitan	2,960,550 (68.1%)	271,680 (71.9%)	839,610 (74.2%)
	Micropolitan	718,380 (16.5%)	56,600 (15.0%)	144,890 (12.8%)
	Small town	412,260 (9.5%)	30,690 (8.1%)	81,250 (7.2%)
	Rural	226,290 (5.2%)	16,320 (4.3%)	48,950 (4.3%)

SVI and RUCA do not total to 100% as those missing appropriate address information are omitted from this table. Other patient sex categories are also omitted.

Source: Electronic health record (EHR) data (2010–2021); 2000, 2010, and 2020 Census; American Community Survey (2005–2022).

Note: The Census Bureau has reviewed this data product to ensure appropriate access, use, and disclosure avoidance protection of the confidential source data used to produce this product (Data Management System (DMS) number: P-7527965, Disclosure Review Board (DRB) approval number: CBDRB‑FY24‑0453). Discrete Gaussian noise was applied to all unweighted counts according to U.S. Census Bureau disclosure protocols to preserve data privacy.

#### iii. Practice-level effects.

There are 1,290 unique primary care practices in the EHR data, and discordance occurs across all practices. We observe no statistically significant relationship between practice size and either discordance rate or missingness rate, meaning that smaller practices, on average, are just as likely to exhibit discordance or missingness as larger practices. Nor do we observe any statistically significant relationship between discordance and missingness rate, meaning that a practice-level observation of low missingness does not imply that discordance is also low. We do, however, observe a positive relationship (p < 0.01) between the heterogeneity of a practice’s patient population (specifically, the share of non-White patients) and both discordance and missingness rate, consistent with our findings in Fig 1 and Table 2.

While discordance appears to be a universal phenomenon across practices, it is possible to identify patterns of discordance that are concentrated in certain practices, suggesting that discordance may be driven by practice-level reporting standards. For instance, 135,810 patients are recorded as non-Hispanic in EHR data but recorded as Hispanic in Census Bureau microdata; 28.4% (38,570/135,810) of these patients come from just ten (out of 1,290) practices. Meanwhile, 125,350 patients are recorded as a single race in EHR data while recorded as Multiracial in Census Bureau microdata; 85.8% (107,550/125,350) of these patients come from large practices (with over 1,000 patients) in which we observe zero Multiracial records—highly unlikely to reflect an actual patient population and surrounding community with no Multiracial individuals.

#### iv. Comparison of ACS and decennial census reporting.

To examine whether the patterns of discordance we observe can be solely explained by idiosyncrasies in the healthcare system, we briefly set aside the EHR-recorded race/ethnicity information and perform a similar analysis for a subset of 270,230 individuals who responded to both the ACS (2019–2022) and the 2020 Census, during which the textual design of the race/ethnicity questions in these two data sources was similar. While the available checkboxes and write-in fields remained the same from 2019 to 2020 and on, some improvements in the design of the race and ethnicity questions [72,73], particularly the inclusion of more examples for certain categories and some checkbox reordering, as well as improvements in the way responses were processed and coded, and other fundamental differences between ACS and decennial census in definitions and methods, may impact direct comparability across these sources and years [74,75]. Fig A in S1 Text visualizes this cohort, comparing race/ethnicity as recorded in the two Census Bureau sources. While groups like NHPI and Multiracial exhibit clearly improved concordance rates, we still find an overall discordance rate of 5.1% (13,780/270,230). This finding is lower than the 8.0% we observed when comparing EHR data to Census Bureau microdata, though accounting for partial concordance brings these values closer to alignment (5.1% versus 5.4%). We note here that while race/ethnicity questions were similar between these two data sources, there were still significant differences in data collection and processing that might affect our conclusions from this comparison. Nevertheless, this analysis suggests that discordance is not solely driven by differences *across* different data collection domains, but rather also exists *within* a single data collection domain.

We also observe discordance within the same type of Census Bureau survey instrument, though these differences may be further driven by explicit changes to the race/ethnicity questions. Out of 4.84 million individuals who answered more than one decennial census in 2000, 2010, or 2020, we find a 7.2% (347,540/4.84M) discordance rate, consistent with prior literature on this topic which found a 6.1% discordance rate between 2000 and 2010 Census responses [10].

### B. Evaluating prevalence of health outcomes in EHR and Census Bureau data

#### i. Sensitivity across racial/ethnic categories.

**Fig 2:**
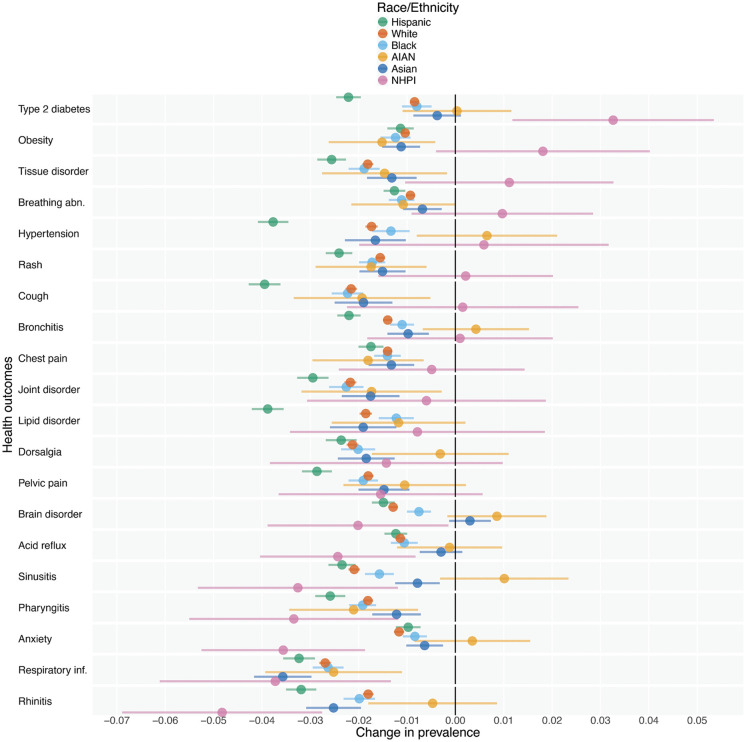
For 20 health outcomes, changes in prevalence estimates for each racial/ethnic group comparing EHR-recorded to Census Bureau-recorded race/ethnicity. The rows represent diagnoses or healthcare events observed in the EHR data, aggregated to the ICD-10 code 3-digit level. Distances along the x-axis represent the percentage point change in outcome prevalence estimates for each racial/ethnic group when switching from EHR-recorded to Census Bureau-recorded race/ethnicity. The error bars represent 95% Wald-type confidence intervals constructed using the delta method, adjusted using a Bonferroni correction with a family-wise error rate of 0.05. The outcomes are sorted based on the NHPI group, for which we see the most substantial changes. The Multiracial group, which experiences larger sensitivities, is omitted for visual clarity (see Fig F in S1 Text). See Table A in S1 Text for full code descriptions. Z codes, which represent reasons for health encounters, are excluded from this subset of top 20 outcomes by prevalence. Source: Electronic health record (EHR) data (2010–2021); 2000, 2010, and 2020 Census; American Community Survey (2005–2022). Note: The Census Bureau has reviewed this data product to ensure appropriate access, use, and disclosure avoidance protection of the confidential source data used to produce this product (Data Management System (DMS) number: P-7527965, Disclosure Review Board (DRB) approval number: CBDRB‑FY24‑0453). Discrete Gaussian noise was applied to all unweighted counts according to U.S. Census Bureau disclosure protocols to preserve data privacy.

Fig 2 illustrates the impact of discordance and missingness on prevalence estimates of 20 health outcomes for 6 racial/ethnic groups (see Appendix D in S1 Text for all 50 outcomes). The NHPI group (by which the outcomes are sorted) experiences the largest increase in disease prevalence out of any group, a 3.3 [1.2-5.3] percentage point shift in Type 2 diabetes when switching from EHR-recorded to Census Bureau-recorded race/ethnicity. It also experiences the largest decrease out of any group, a 4.8 [2.8-6.9] percentage point shift in rhinitis prevalence. Other groups also experience large substantive downward shifts in disease prevalence when switching to Census Bureau-recorded race/ethnicity, such as the Hispanic group experiencing nearly 4 percentage point reductions in hypertension, cough, and lipid disorder prevalence.

#### ii. A closer look at type 2 diabetes.

**Fig 3:**
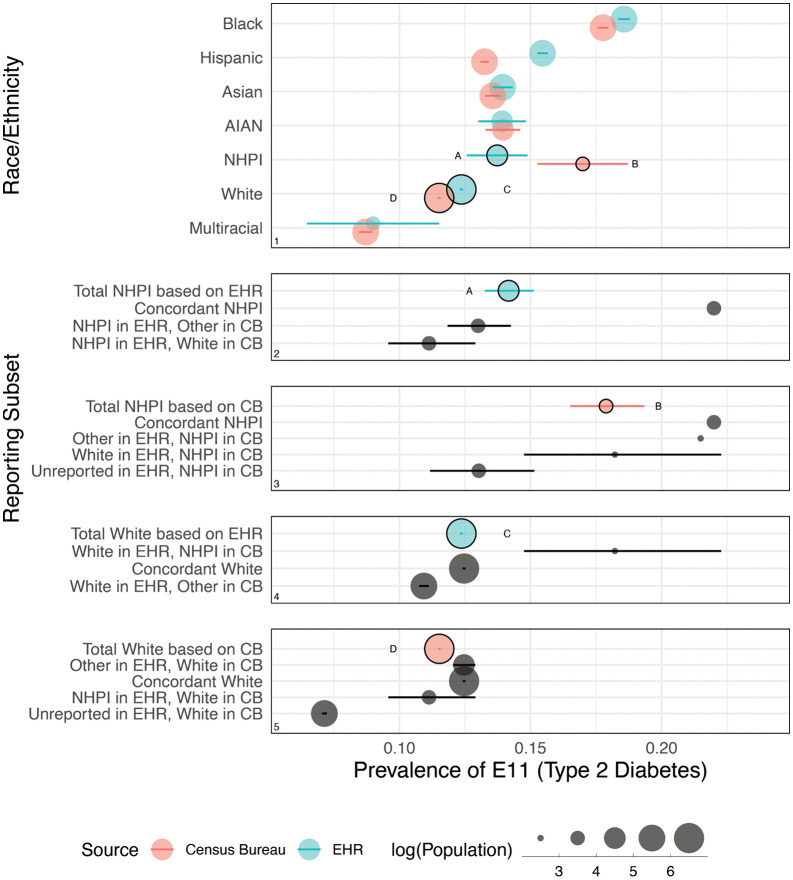
(Panel 1) Prevalence of Type 2 diabetes by race/ethnicity, as recorded in Census Bureau data versus EHR data. (Panels 2-5) Disaggregation of estimates (A-D) to different constituent patient subsets, as distinguished by recording patterns. In Panel 1, the rows are ordered from highest to lowest absolute prevalence, as calculated using EHR data (blue) and Census Bureau (CB) microdata (red). In Panels 2–5, the gray points represent subsets of patients (some of which show up in multiple panels) which add up to the racial/ethnic group represented by the colored point. The error bars represent 95% confidence intervals constructed using the Wilson Score method (with continuity correction), adjusted using a Bonferroni correction with a family-wise error rate of 0.05. Circle sizes represent population in base-10 log scale. Estimates subject to disaggregation in panels 2–5 are identified with circle outlines and labels (A-D). Minor differences between panels (i.e., the prevalence values of A-D) are due to disclosure-required noise infusion. Source: Electronic health record (EHR) data (2010–2021); 2000, 2010, and 2020 Census; American Community Survey (2005–2022). Note: The Census Bureau has reviewed this data product to ensure appropriate access, use, and disclosure avoidance protection of the confidential source data used to produce this product (Data Management System (DMS) number: P-7527965, Disclosure Review Board (DRB) approval number: CBDRB‑FY24‑0453). Discrete Gaussian noise was applied to all unweighted counts according to U.S. Census Bureau disclosure protocols to preserve data privacy.

We highlight one particular condition, Type 2 diabetes (the first health outcome in Fig 2), for further illustration in Fig 3 (a similar plot showing all 50 outcomes is provided in Fig B in S1 Text), and disaggregate the patterns of concordance, discordance, and missingness that shape changes in estimated group-level prevalences. Notably, comparing EHR-recorded to Census Bureau-recorded race/ethnicity in Panel 1 results in an increase in NHPI prevalence (A to B) from 13.7% [12.6-14.9] to 17.0% [15.3-18.7], but decrease in White prevalence (C to D) from 12.4% [12.3-12.4] to 11.5% [11.4-11.6]. These changes in prevalence estimation also amount to changes in estimations of differences across groups. For example, NHPI-White difference per EHR recorded race/ethnicity (A minus C) is 1.4 [-0.3-3.0] percentage points, but per Census Bureau-recorded race/ethnicity (B minus D) is 5.5 [3.0-7.9] percentage points.

Panels 2–5 illustrate how specific cohorts of concordance, discordance, and missingness shape differences in prevalence of Type 2 diabetes. Comparing the two versions of NHPI prevalence in Panels 2 and 3, (A) and (B), those who are only recorded as NHPI in EHR data (but are recorded differently in Census Bureau microdata) have relatively lower prevalence, thereby pulling the total EHR-observed NHPI prevalence down. Notably, those who are fully concordant in their recording as NHPI, *i.e.*, those we may most confidently conclude to be NHPI, have the highest prevalence of Type 2 diabetes (over 20%), which is masked by the lower prevalence of discordantly recorded or nonreporting patients. Moving over to comparing the two versions of White prevalence in Panels 4 and 5, (C) and (D), the subset driving the Census Bureau prevalence down are patients who were missing race/ethnicity in EHR data but were recorded as White in Census Bureau microdata (last row of Panel 5). The Type 2 diabetes prevalence for nonreporting White patients is 7.1% whereas the prevalence for nonreporting NHPI patients is nearly twice as high at 13.0% (last row of Panel 3). These two nonreporting groups do not impact the observed NHPI or White Type 2 diabetes prevalences in EHR data, but once their race/ethnicity information is enhanced with Census Bureau microdata, they contribute to a widened diabetes gap.

## 5. Discussion

The linkage of EHR data and Census Bureau microdata yielded 5.86 million (93.9%) matched individuals, for whom we identified notable differences in recorded race/ethnicity. While the scale of missingness in EHR data is observable just by inspecting EHR data themselves, the linkage enabled us to characterize that missingness across racial and ethnic groups, in particular finding that Asian and NHPI patients exhibit substantially higher missingness than other groups. Through comprehensive record linkage, we were also able to quantify and characterize discordance between administrative sources, finding a discordance rate of 8.0% overall (5.4% if allowing for partial concordance for the Multiracial group) and particularly high discordance among the smallest groups. These patterns corroborate similar findings for linkages between health and other administrative data sources in other studies, both within [76,77] and outside [78] the U.S. We found suggestive evidence of practice-level effects on discordance, particularly that Hispanic discordance may be driven by the lack of an ethnicity category option, and that Multiracial discordance may be driven by the inability for respondents to select multiple race options (*i.e.*, thereby resorting to a single race selection which is by and large consistent with part of their Multiracial identity, as we observed in our partial concordance analysis). These two mechanisms, in effect, inhibit the selection of non-White categories and contribute to the appearance of a less racially and ethnically diverse patient population in EHR data. At the same time, we confirmed that discordance cannot solely be explained by the domain differences between healthcare and government reporting, finding similar patterns of discordance even between relatively contemporaneous Census Bureau data sources. Finally, we illustrated the nontrivial impact that the choice of administrative data source can have on data quality and accurate prevalence estimation, using the healthcare system itself as an example. Across the most common health outcomes, we found substantial exacerbations and ameliorations of prevalence differences between groups. Below, we further discuss four key implications of these findings.

### A. The existence of a gold standard

First, our study supports prior findings that call into question the existence of a “gold standard” source of administrative information about race and ethnicity [12,62]. In particular, this is evidenced by our finding of 5.1% to 7.2% discordance even within repeat responses to similarly designed Census Bureau instruments. Thus, in our primary analysis comparing EHR-recorded and Census Bureau-recorded race/ethnicity, while we framed our interpretations of concordance and discordance from the standpoint of Census Bureau-recorded race/ethnicity, we refrained from describing the Census Bureau result as “correct” or “ground truth”, and underscore that neither data source perfectly captures racial/ethnic identity; for any particular patient, the response they ultimately consider to be “correct” could be one or the other, or neither, or both (*e.g.*, fluid across time). While some explanations are well-understood, such as the reporting of race and ethnicity at the household level in ways that may differ from self-reports, and changes to the wording of race and ethnicity questions, our findings may suggest the existence of some base rate of racial fluidity and underscore the socially constructed nature of race. As OMB has recently revised its standards on how federal agencies are required to collect race and ethnicity data, including disaggregation of existing categories, incorporation of the Hispanic category into a combined race/ethnicity question, and the creation of a new Middle Eastern and North African category [58], future work should investigate how race and ethnicity reporting to the Census Bureau evolves, and whether those patterns trickle down to race and ethnicity reporting in healthcare and other consequential domains. As we have seen from past changes to OMB standards, healthcare practices do not adapt their EHR systems to new standards at similar rates, creating challenges for comparability of population health assessments, given the intermittent frequency of patient information updates.

### B. Intra-domain versus inter-domain discordance

Second, our study introduces a distinction between different potential levels of discordance. Most efforts to measure discordance focus on the *intra*-domain level, *i.e.*, comparing multiple observations of an individual’s information within the same information collection domain. We contribute corroborative evidence of this level of discordance in our results focused on discordance across different Census Bureau instruments. However, through intensive record linkage efforts, we are also able to assess *inter*-domain discordance between two widely different settings: one a decentralized and largely private network of healthcare facilities, the other a federal statistical system. This raises a key question: are the two levels of discordance estimates we identified in our study—8.0% between EHR data and Census Bureau microdata, versus 5.1% between ACS (2019–2022) and decennial census microdata (2020 Census)—distinct or overlapping phenomena? As previously noted, accounting for partial concordance of Multiracial records brings the 8.0% estimate down to a more aligned 5.4%. This consilience of estimates—and the finding that partial concordance may be largely driven by practice-level errors in recording Multiracial identities—suggests a possible interpretation that roughly 3% of discordance may be more procedural or technological in nature, compared to roughly 5% of discordance that may be more reflective of a fundamental racial fluidity. On the other hand, these two discordance estimates may be far more mutually exclusive. Given the observed downstream impacts on disease prevalence estimates, our study underscores the importance of considering a fuller account of discordance across the many administrative traces of an individual’s racial and ethnic identity.

### C. Methods for discordance researchers

Third, we provide a framework in which researchers can continue to investigate discordance and its impact on downstream analyses and prediction tasks in fields such as statistics and machine learning. Given the ability for record linkage across two generally reputable sources like EHR data [22] and Census Bureau microdata, researchers can conduct analyses of one reporting source conditional on what is reported in the other source, and vice versa, to surface particular patterns of discordance. For example, our focused examination of the subset of patients who were recorded as White in EHR data but differently in Census Bureau microdata led to the discovery that discordance is most common for an outlier set of practices; such insights may yield practice-level reforms that ensure all race and ethnicity options are accessible (*i.e.*, a Hispanic option, or the ability to select multiple race options), or reduce the chance of patients unintentionally misreporting race and ethnicity, or discourage healthcare practitioners from filling in race and ethnicity on behalf of patients. Given particular outcomes of interest, such as health diagnoses in our case, researchers can also compare outcomes across sub-populations based on reporting patterns, as we demonstrate in Fig 3, to uncover possible correlations of discordance with the outcome itself. While our case study of Type 2 diabetes provides some suggestive corroboration of Saperstein [62]—who argues that individuals who experience an increase in social mobility may change their racial and ethnic identification as a result of such mobility, and vice versa—in the domain of health status, our observational study does not enable a more conclusive causal inference.

### D. Actionable techniques for population health practitioners

Fourth, we believe the sensitivity of demographic insights to the combined effects of missingness and discordance has significant implications for any institution that takes corrective action, or not, based on one available source of demographic attributes and outcomes. While our clearest examples of practitioners in this study are those in population health, or those making use of Census Bureau statistical products, we argue these implications extend to many other settings. In situations where record linkage is available, similar to our study, some options include incorporating multiple sources of information into a formal sensitivity analysis, or treating race as a latent variable with measurement error. An outcome that is corroborated by multiple sources (*e.g.*, our finding that those who were concordantly recorded as NHPI had a substantially higher prevalence of Type 2 diabetes) may inspire more immediate action, whereas an outcome that is only partially evidenced may inspire audits of data quality before any further action.

In situations where record linkage is unavailable, practitioners may consider prioritizing in-house audits to uncover potential drivers of missingness or discordance in demographic information, such as outdated forms or protocols. However, both the audits themselves and the internal quality improvements informed by those audits may incur substantial time and resource costs for individual facilities or agencies [79], and may help reduce but likely not eliminate the actual problems of missingness and discordance. A more cost-effective alternative may be imputation of demographics using other individual attributes and statistical or machine learning methods [80]. Despite being susceptible to some degree to the same underlying discordance issues, imputation can nonetheless rigorously incorporate uncertainty estimation [81], leading to the incorporation of confidence intervals in group-level assessments. Imputation methods like Bayesian Improved Surname Geocoding [31], which are based on conditional probabilities of race and ethnicity, name, and geography sourced from external administrative sources, may serve as a promising and accessible diagnostic test for the most egregious forms of discordance attributable to practice-level errors. For example, if individual practices have erroneous data collection and processing procedures that lead to the complete absence or rare recording of Hispanic or Multiracial patients, as we observed in our study, such errors may be readily identifiable based on the unusually high discordance between practice labels and BISG-predicted labels, and practices would have all the information and tools necessary to conduct such internal audits.

At a minimum, we hope that an awareness and appreciation of the potential for demographic reporting and recording differences, some base rate fluidity of racial and ethnic identification, and the possibility that these heterogeneities may affect data quality lead to more rigor in, and long-term refinement of, group-level assessments.

### E. Limitations and future work

We now discuss methodological limitations and how future efforts may build upon our work. First, while EHR data are shown to have high informational value, they are likely biased in their racial and ethnic composition and quality of information through multiple mechanisms, including differential coverage across the U.S., differential reporting protocols across primary care practices and EHR systems (including the possibility that information is not self-reported), and differential care-seeking behavior across groups and conditions [82]. Therefore, we do not interpret any of the EHR-wide distributions of demographics to be representative of the U.S. population. While we use EHR data linked to Census Bureau microdata, which itself more comprehensively covers the U.S., we do not use Census Bureau microdata to improve the representativeness of EHR data. Instead, our study reflects a practical setting in which the given administrative dataset represents the complete universe within which observed health prevalences matter to specific stakeholders and shape corrective actions. For example, our EHR data are derived from a national network of primary care physicians who provide care specifically to the patients documented within the dataset, and for whom awareness (or not) of population health concerns may cause the direct implementation (or not) of specific policies and programs at the clinic and/or patient level. Therefore, we do not make any attempt to alter the composition of our EHR data. Future work could corroborate the patterns of missingness and discordance we find across other pairs of administrative records in different domains (*e.g.*, education, law enforcement, voter rolls), as well as in administrative systems outside the U.S.

Second, while we attempted to harmonize race and ethnicity coding as much as possible between our two sources, EHR data and Census Bureau microdata have fundamental differences, some of which we are unable to examine, that may contribute to some portion of the base discordance we observe. We conducted random audits of the raw EHR fields of race and ethnicity information to confirm that the final group mapped to 1997 OMB standards is generally well-aligned with the underlying source data. The need for harmonization across EHR systems and practices, of course, itself reflects a baseline heterogeneity of reporting standards that, at minimum, complicates efforts to understand and improve quality of care at scale. For either data source, we are unable to distinguish whether race/ethnicity information (and other information like sex) are being self-reported by the individual or reported by someone else (*e.g.*, another member of the household for the decennial census, or healthcare staff for EHRs), the latter of which could contribute in some way to discordance. We also cannot definitively determine whether any observed discordance between two distinct singular racial/ethnic identities for an individual is to be interpreted as such, or is actually partial reporting of an overall Multiracial identity, which would affect the specific patterns we present but not the underlying existence of a discordance problem. In general, the greater the temporal mismatch between the latest available EHR and Census Bureau records for any particular individual, the greater the opportunity for our discordance measure to capture actual racial fluidity as distinct from technological drivers of discordance, which our study is unable to disentangle. Lastly, PIK assignment in the Census Bureau record linkage process is partially probabilistic and inherently imperfect, and, along with the discrete Gaussian noise applied to all unweighted counts in our analysis, may account for some degree of the discordance we observe. Overall, our results still point conclusively to the existence of discordance, and to the nontrivial effects of both missingness and discordance on population health assessments.

Third, while we were limited in our ability to investigate more practice-level drivers of discordance and missingness, given the terms of use of our unique linked research environment, future work could extend our analysis through multi-level modeling and investigation of information intake interfaces and other practice-level attributes. Both researchers and practitioners could identify, document, and correct specific instances that corroborate the alarming signals we discovered of practices entirely missing Hispanic or Multiracial records, as well as other procedural or technological drivers of discordance beyond the scope of our study. Future work could also examine more granular health outcomes available in EHR data, such as more detailed versions of ICD-10 codes, medical procedure codes, and unstructured information within clinical notes.

## 6. Conclusion

We demonstrate how a novel data linkage strategy can be leveraged to thoroughly assess data quality, specifically patterns of missingness and the less-understood phenomenon of discordance, and to more accurately estimate disease prevalence. By strategically repurposing administrative records through linkages typically not available to researchers, we comprehensively document that discordance is both widespread and highly concentrated in specific administrative settings, creating risk of bias at every geographic scale of assessment. However, as we demonstrate through our evaluation, much can be done in real-world settings with generalizable investigative techniques to diagnose and mitigate discordance and missingness, particularly data quality improvement at the point when demographic information is collected. Ultimately, with more complete and concordant demographic information and improved data quality in electronic health records and other administrative records, government agencies, academics, and practitioners can more accurately measure and address health challenges.

## Supporting information

S1 Text Supporting information. Contains Appendices A through D.
(PDF)
