# Supplementary material for: Evaluating the impact of discordant and missing demographic information on population health assessments using linked electronic health records and Census Bureau microdata
Source: PLOS Digit Health. 2026 Mar 17;5(3):e0001289. doi: 10.1371/journal.pdig.0001289 (PMC12994837; doi:10.1371/journal.pdig.0001289)
Supplement: S1 Text — Contains Appendices A through D. (PDF) [file pdig.0001289.s001.pdf]

Supporting Information for: Evaluating the impact of discordant and missing demographic information on population health assessments using linked electronic health records and Census Bureau microdata

A. Race and ethnicity variable construction

Race and ethnicity are collected separately in the EHR data. For race, there are two fields for each patient: *patientracecode* and *patientracetext*, consolidated from all practices. *patientracecode* may contain one or more parent codes (*e.g.*, 1002-5 for AIAN) and/or child codes (*e.g.*, 1010-8 for Apache) matching the official Health Level 7 (HL7) codes established by the U.S. Centers for Disease Control and Prevention (CDC). *patientracetext* contains free text. The dataset exhibits wide heterogeneity in the consistency and clarity of provider data standards, ranging from records with codes and code descriptions that always exactly match the CDC system, to records with no code and only single characters of free text.

A derived race variable was constructed using the following steps. First, a string match was sought between any code in *patientracecode* and any Level 1 HL7 category, and between any subset of terms of *patientracetext* and any text description within the category, including some spelling variations. The terms "caucasian," "wh," and "w," among others, were considered text matches to White. The terms "blk," "b," and "aa," among others, were considered text matches to Black. Second, patients were assigned a racial category based on code matches and another based on text matches, where matches in more than one category were assigned Multiracial. Third, a final racial variable was assigned, prioritizing the code-based match over the text-based match. If *patientracecode* matched 2131-1 for Other Race, but *patientracetext* matched to a racial category, the text-based match was used. The remaining patients, who

have neither a code-based or text-based match, including text descriptions such as “unknown,” “decline,” or “refused,” were assigned Unknown.

A similar approach was used to derive an ethnicity variable from *patientethnicitycode* and *patientethnicitytext*. The terms “hispanic,” “latino,” “hispa,” “his,” or “h,” among others, were considered text matches to Hispanic. We excluded the following regex matches: “not-hispanic”, “not hispanic”, “non-hispanic”, “non - hispanic”, “non hispanic”, “not hispanic or latino”. Any code-based match, or text-based match if there was no code-based match, to Hispanic led to the final assignment of Hispanic. All other patients were assigned non-Hispanic, or Unknown.

A final race/ethnicity variable was constructed for both EHR and Census Bureau data sources which combined race and ethnicity into one category. If ethnicity was Hispanic, then the combined race/ethnicity was assigned Hispanic, regardless of race (even if unknown). If ethnicity was non-Hispanic, or Unknown, then the combined race/ethnicity was assigned the value of the race variable. If ethnicity was non-Hispanic, or Unknown, and race was Unknown, then the combined race/ethnicity was assigned Unknown.

B. Health outcomes codes information

ICD-code, 3 digits	Shorthand	Description
E03	Hypothyroidism	Other hypothyroidism
E11	Type 2 diabetes	Type 2 diabetes mellitus
E55	Vit. D deficit	Vitamin D deficiency
E66	Obesity	Overweight and obesity
E78	Lipid disorder	Disorders of lipoprotein metabolism and other lipidemias
F32	Depression	Depressive episode
F41	Anxiety	Other anxiety disorders
G44	Headaches	Other headache syndromes
G47	Sleep disorder	Sleep disorders
G93	Brain disorder	Other disorders of brain
H66	Ear infection	Suppurative and unspecified otitis media
H92	Ear pain	Otalgia and effusion of ear
I10	Hypertension	Essential (primary) hypertension
J01	Sinusitis	Acute sinusitis
J02	Pharyngitis	Acute pharyngitis
J06	Respiratory inf.	Acute upper respiratory infections of multiple and unspecified sites
J20	Bronchitis	Acute bronchitis
J30	Rhinitis	Vasomotor and allergic rhinitis
J45	Asthma	Asthma
K21	Acid reflux	Gastro-esophageal reflux disease
K52	Gastroenteritis	Other and unspecified noninfective gastroenteritis and colitis
K59	Intestinal dis.	Other functional intestinal disorders
L03	Lymphangitis	Cellulitis and acute lymphangitis
M25	Joint disorder	Other joint disorder, not elsewhere classified
M54	Dorsalgia	Dorsalgia

M79	Tissue disorder	Other and unspecified soft tissue disorders, not elsewhere classified
N30	Cystitis	Cystitis
N39	Urinary dis.	Other disorders of urinary system
R05	Cough	Cough
R06	Breathing abn.	Abnormalities of breathing
R07	Chest pain	Pain in throat and chest
R10	Pelvic pain	Abdominal and pelvic pain
R11	Nausea	Nausea and vomiting
R19	Digestive iss.	Other symptoms and signs involving the digestive system and abdomen
R21	Rash	Rash and other nonspecific skin eruption
R30	Urinary pain	Pain associated with micturition
R42	Dizziness	Dizziness and giddiness
R50	Fever	Fever of other and unknown origin
R53	Fatigue	Malaise and fatigue
R63	Food intake iss.	Symptoms and signs concerning food and fluid intake
R73	Hyperglycemia	Elevated blood glucose level
Z00	General exam	Encounter for general examination without complaint, suspected or reported diagnosis
Z01	Special exam	Encounter for other special examination without complaint, suspected or reported diagnosis
Z11	Inf. dis. screen	Encounter for screening for infectious and parasitic diseases
Z12	Cancer screen	Encounter for screening for malignant neoplasms
Z13	Other screen	Encounter for screening for other diseases and disorders
Z23	Immunization	Encounter for immunization
Z68	BMI measure	Body mass index [BMI]
Z71	Counseling	Persons encountering health services for other counseling and medical advice, not elsewhere classified
Z79	Drug therapy	Long term (current) drug therapy

Table A. Top fifty health outcomes, collapsed to the first 3 digits of ICD-10 codes, for patients in our study cohort.
Source: Electronic health record (EHR) data (2010-2022)

C. Additional discordance and missingness results

Decennial response (columns) × ACS response (rows)

Counts

ACS response	Hispanic	White	Black	AIAN	Asian	NHPI	Multiracial	Total
Hispanic	16,780	1,530	90	160	20	20	140	18,740
White	1,580	212,650	190	320	150	30	3,300	218,220
Black	160	250	14,580	130	<15	<15	300	15,410
AIAN	<15	330	<15	1,220	<15	<15	410	1,960
Asian	30	440	<15	<15	7,180	40	150	7,850
NHPI	<15	100	<15	20	<15	140	20	290
Multiracial	60	2,880	290	380	140	110	3,910	7,770
Total	18,600	218,180	15,150	2,230	7,500	350	8,220	270,230

Percent of Total by Row

ACS response	Hispanic	White	Black	AIAN	Asian	NHPI	Multiracial
Hispanic	89.6%	8.1%	0.5%	0.8%	0.1%	0.1%	0.7%
White	0.7%	97.4%	0.1%	0.1%	0.1%	0%	1.5%
Black	1%	1.6%	94.6%	0.9%	NA	NA	1.9%
AIAN	NA	17%	NA	62.2%	NA	NA	20.8%
Asian	0.4%	5.6%	NA	NA	91.5%	0.5%	1.9%
NHPI	NA	35.9%	NA	7.2%	NA	50%	6.9%
Multiracial	0.7%	37%	3.8%	4.8%	1.8%	1.4%	50.4%
Total	6.9%	80.7%	5.6%	0.8%	2.8%	0.1%	3%

Percent of Total (excl. Unreported) by Column

ACS response	Hispanic	White	Black	AIAN	Asian	NHPI	Multiracial	Total
Hispanic	90.2%	0.7%	0.6%	7%	0.3%	7.1%	1.7%	6.9%
White	8.5%	97.5%	1.2%	14.6%	2%	8.5%	40.1%	80.8%
Black	0.8%	0.1%	96.2%	5.9%	NA	NA	3.6%	5.7%
AIAN	NA	0.2%	NA	54.7%	NA	NA	5%	0.7%
Asian	0.2%	0.2%	NA	NA	95.9%	12.1%	1.8%	2.9%
NHPI	NA	0%	NA	0.9%	NA	41%	0.2%	0.1%
Multiracial	0.3%	1.3%	1.9%	16.9%	1.9%	31.4%	47.6%	2.9%

Legend: Concordant; Discordant; Total; Percent

Fig A. Matrix comparing race/ethnicity as recorded in the 2020 Census (grouped by column) versus race/ethnicity as recorded in the American Community Survey (ACS, 2019-2022; grouped by row), for 270,230 individuals. (Upper left) Counts of individuals. (Upper right) Percentages which sum to 100 by row and represent the distribution of those individuals in an ACS-recorded racial/ethnic group as recorded in decennial census microdata. (Bottom left) Percentages which sum to 100 by column and represent the distribution of those individuals in a decennial census-recorded racial/ethnic group as recorded in ACS microdata. Counts less than 15, and related proportions, are suppressed (NA). Color scales are for illustration only.
Source: Electronic health record (EHR) data (2010-2022); 2020 Census; American Community Survey (2019-2022).
Note: The Census Bureau has reviewed this data product to ensure appropriate access, use, and disclosure avoidance protection of the confidential source data used to produce this product (Data Management System (DMS) number: P-7527965, Disclosure Review Board (DRB) approval number: CBDRB-FY24-0453). Discrete Gaussian noise was applied to all unweighted counts according to U.S. Census Bureau disclosure protocols to preserve data privacy.

D. Additional health outcome results

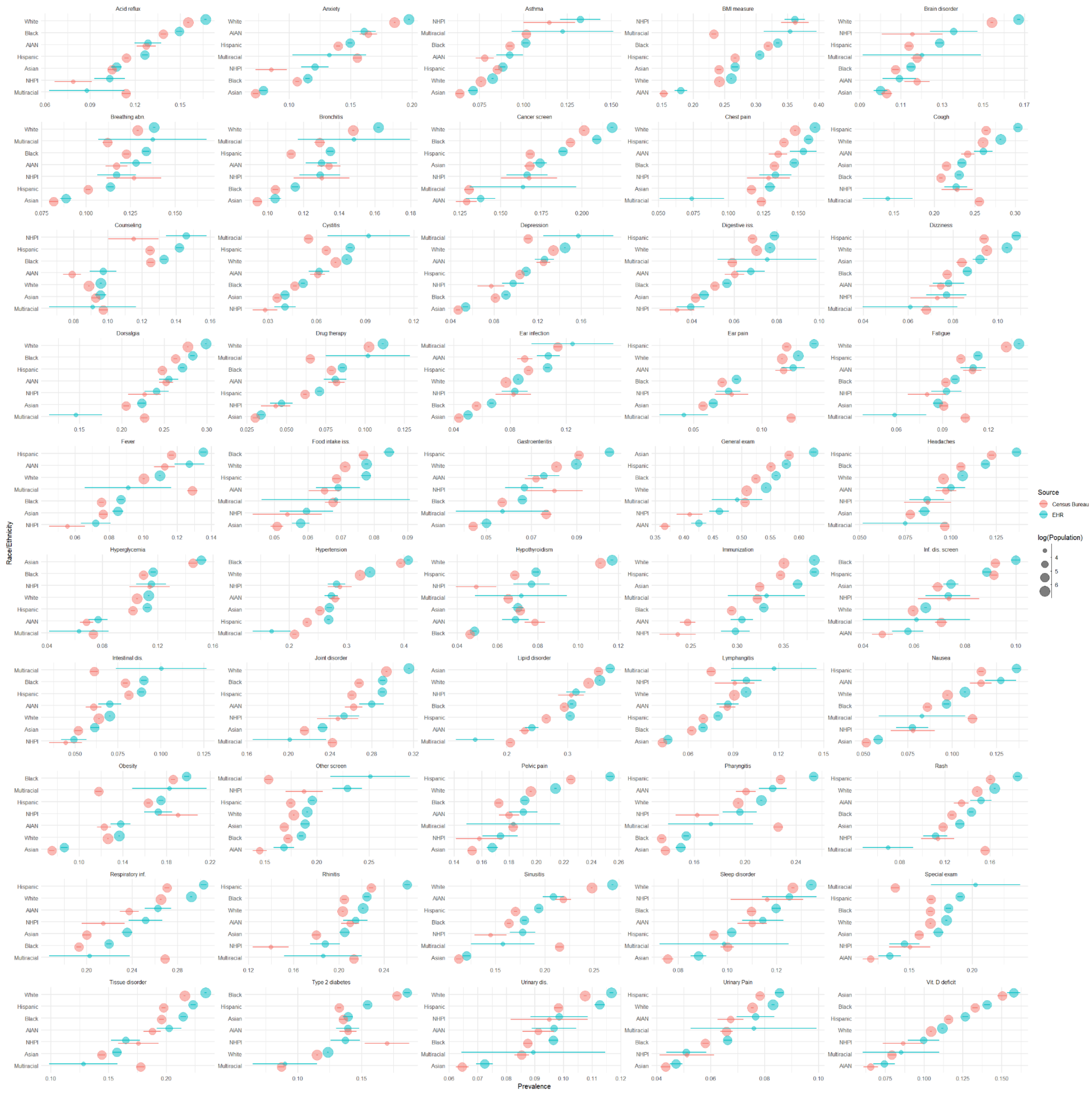

Fig B. Prevalence of the top 50 health outcomes by race/ethnicity, as reported to the Census Bureau (red) versus as reported in EHR data (blue). See Table A for the ICD codes and full descriptions corresponding to shorthand diagnosis labels. See Figs C and D for alternative versions in which we adjust the labels of 120,860 patients who are partially concordant in their reporting of Multiracial.
Source: Electronic health record (EHR) data (2010-2022); 2000, 2010, and 2020 Census; American Community Survey (2001-2022).
Note: The Census Bureau has reviewed this data product to ensure appropriate access, use, and disclosure avoidance protection of the confidential source data used to produce this product (Data Management System (DMS) number: P-7527965, Disclosure Review Board (DRB) approval number: CBDRB-FY24-0453). Discrete Gaussian noise was applied to all unweighted counts according to U.S. Census Bureau disclosure protocols to preserve data privacy.

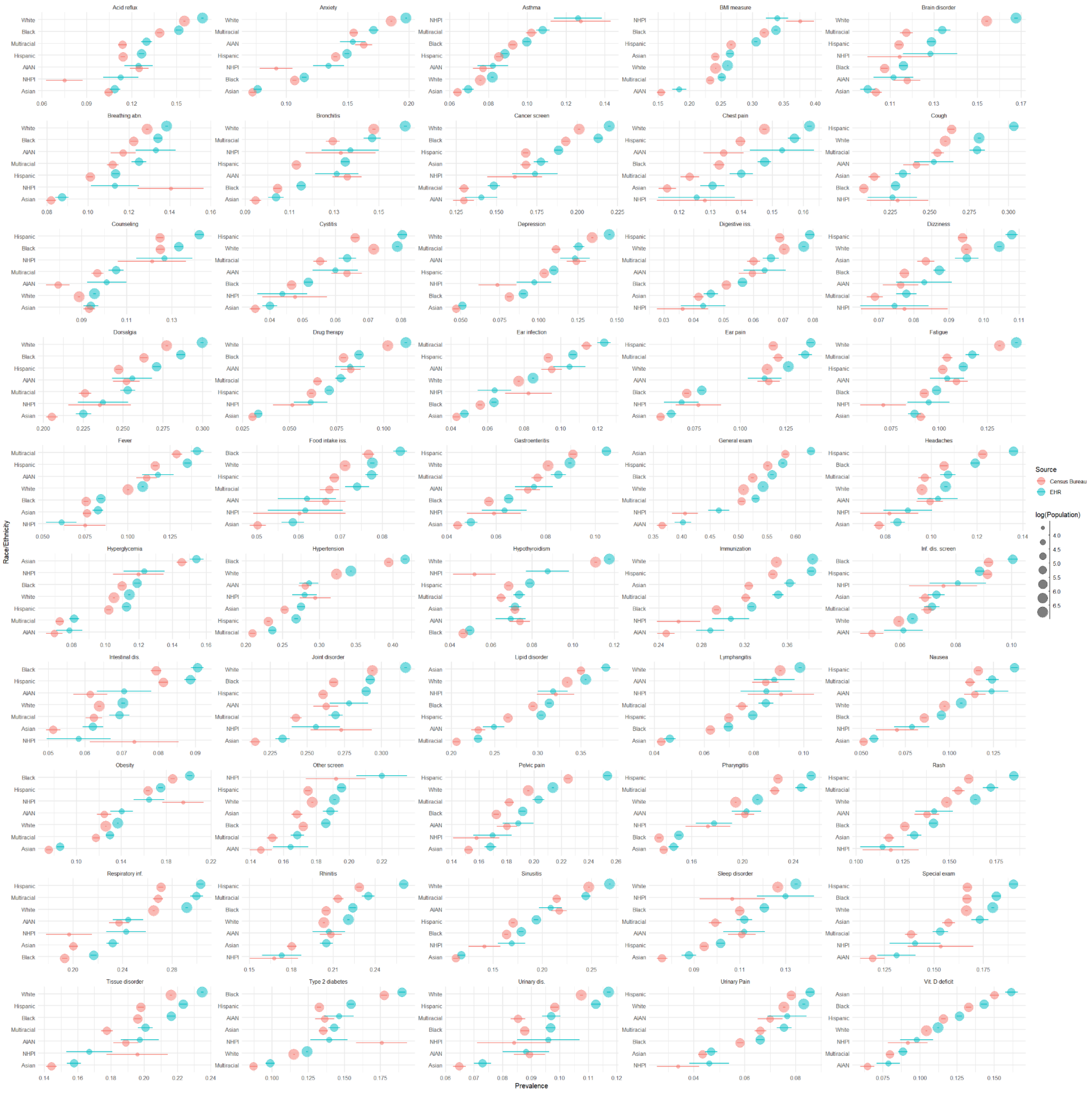

Fig C. Prevalence of the top 50 health outcomes by race/ethnicity, as reported to the Census Bureau (red) vs. as reported in EHR data (blue). In this version, for 120,860 patients who report Multiracial in one source but not in the other, but are partially concordant, we set both labels as concordantly Multiracial. The primary effect is to increase the number of patients who appear to report Multiracial in EHR data (blue). See Table A for the ICD codes and full descriptions corresponding to shorthand diagnosis labels.

Source: Electronic health record (EHR) data (2010-2022); 2000, 2010, and 2020 Census; American Community Survey (2001-2022).

Note: The Census Bureau has reviewed this data product to ensure appropriate access, use, and disclosure avoidance protection of the confidential source data used to produce this product (Data Management System (DMS) number: P-7527965, Disclosure Review Board (DRB) approval number: CBDRB-FY24-0453). Discrete Gaussian noise was applied to all unweighted counts according to U.S. Census Bureau disclosure protocols to preserve data privacy.

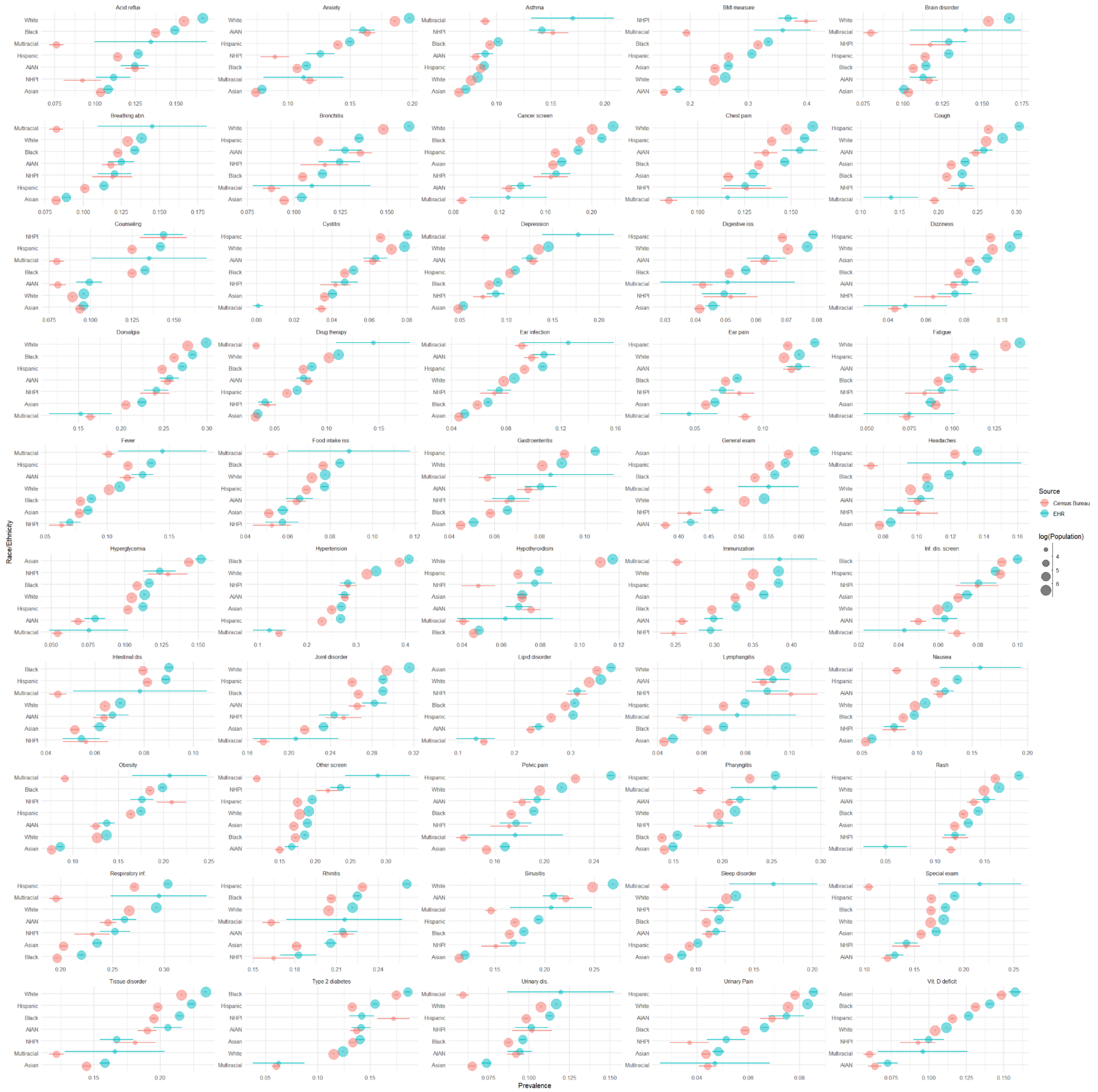

Fig D. Prevalence of the top 50 health outcomes by race/ethnicity, as reported to the Census Bureau (red) vs. as reported in EHR data (blue). In this version, for 120,860 patients who report Multiracial in one source but not in the other, but are partially concordant, we set both labels as the single race that was concordant. The primary effect is to decrease the number of patients who appear to report Multiracial in Census Bureau microdata (red). See Table A for the ICD codes and full descriptions corresponding to shorthand diagnosis labels.

Source: Electronic health record (EHR) data (2010-2022); 2000, 2010, and 2020 Census; American Community Survey (2001-2022).

Note: The Census Bureau has reviewed this data product to ensure appropriate access, use, and disclosure avoidance protection of the confidential source data used to produce this product (Data Management System (DMS) number: P-7527965, Disclosure Review Board (DRB) approval number: CBDRB-FY24-0453). Discrete Gaussian noise was applied to all unweighted counts according to U.S. Census Bureau disclosure protocols to preserve data privacy.

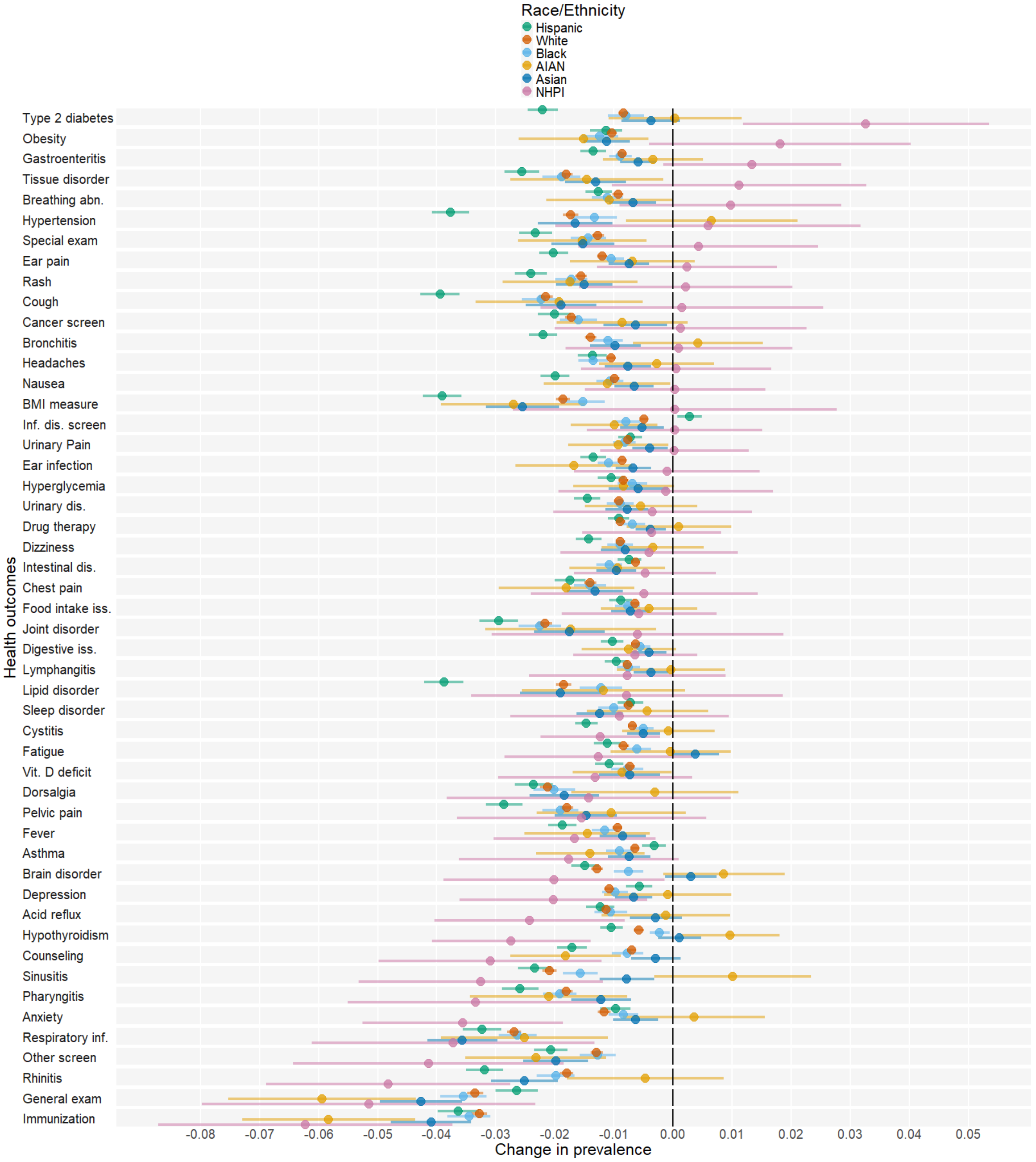

Fig E. For the top 50 health outcomes, changes in prevalence estimates for each racial/ethnic group when switching from EHR-recorded to Census Bureau-recorded race/ethnicity. The Multiracial group, which experiences larger sensitivities, is omitted for visual clarity (see Fig F). The error bars represent 95% Wald-type confidence intervals constructed using the delta method, adjusted using a Bonferroni correction with a family-wise error rate of 0.05. See Table A in S1 Text for full code descriptions.

Source: Electronic health record (EHR) data (2010-2022); 2000, 2010, and 2020 Census; American Community Survey (2001-2022).

Note: The Census Bureau has reviewed this data product to ensure appropriate access, use, and disclosure avoidance protection of the confidential source data used to produce this product (Data Management System (DMS) number: P-7527965, Disclosure Review Board (DRB) approval number: CBDRB-FY24-0453). Discrete Gaussian noise was applied to all unweighted counts according to U.S. Census Bureau disclosure protocols to preserve data privacy.

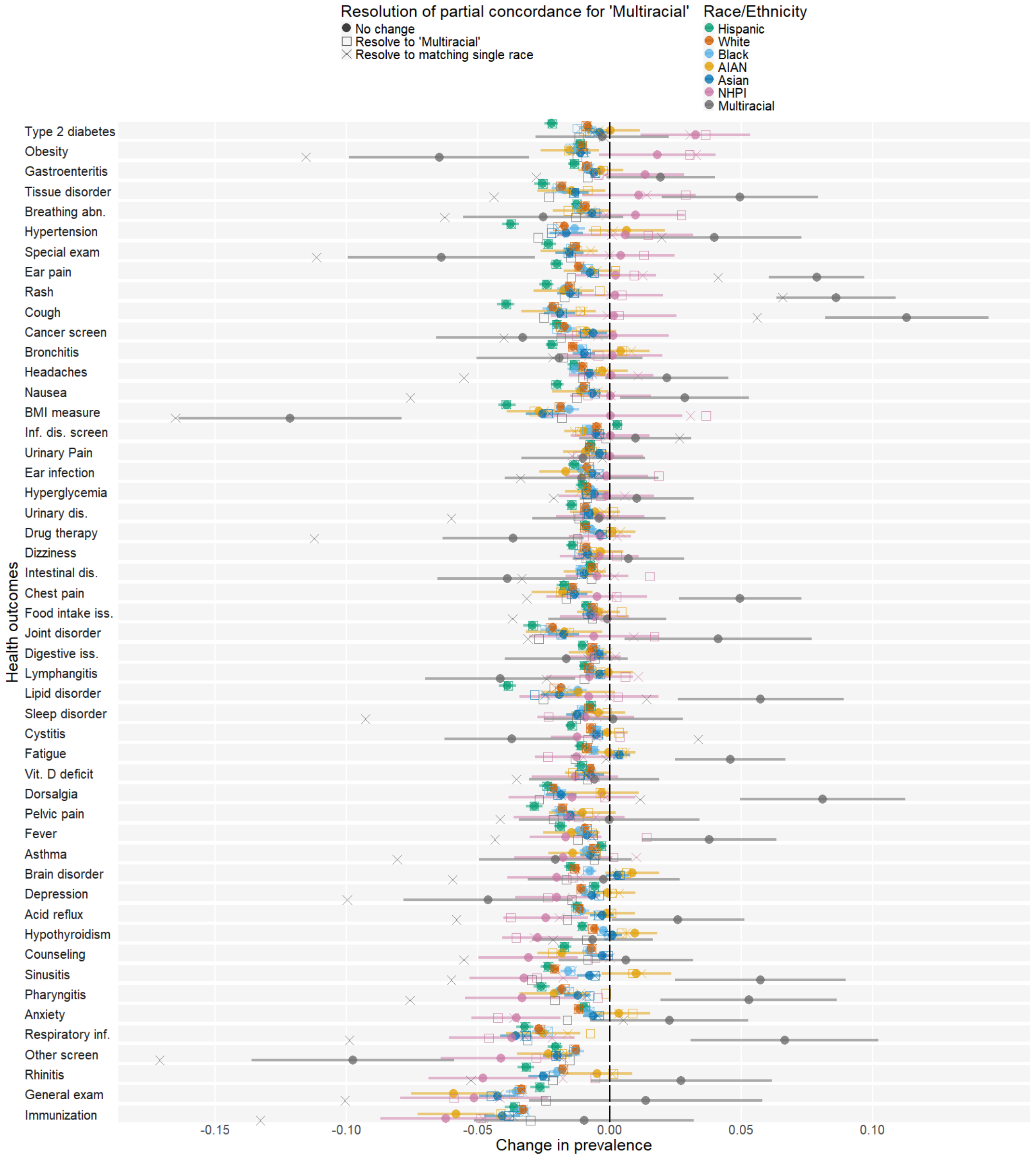

Fig F. For the top 50 health outcomes, changes in prevalence estimates for each racial/ethnic group when switching from EHR-recorded to Census Bureau-recorded race/ethnicity. The error bars represent 95% Wald-type confidence intervals constructed using the delta method, adjusted using a Bonferroni correction with a family-wise error rate of 0.05. The solid points and error bars are the same as shown in Fig 2. This figure includes Multiracial, and includes two additional points for each outcome, which represent two alternative ways to resolve the labels of 120,860 patients who report Multiracial in one source but not in the other, but are partially concordant. For the square points, we set both labels as concordantly Multiracial. For the cross points, we set both labels as the single race that was concordant. Error bars for these alternatives are omitted for clarity. See Table A for the ICD codes and full descriptions corresponding to shorthand diagnosis labels.
Source: Electronic health record (EHR) data (2010-2022); 2000, 2010, and 2020 Census; American Community Survey (2001-2022).
Note: The Census Bureau has reviewed this data product to ensure appropriate access, use, and disclosure avoidance protection of the confidential source data used to produce this product (Data Management System (DMS) number: P-7527965, Disclosure Review Board (DRB) approval number: CBDRB-FY24-0453). Discrete Gaussian noise was applied to all unweighted counts according to U.S. Census Bureau disclosure protocols to preserve data privacy.
